# Scaling Early Detection for Developmental Dysplasia of the Hip With Artificial Intelligence-Assisted Imaging

**DOI:** 10.7759/cureus.102507

**Published:** 2026-01-28

**Authors:** Ibrahim D Al-Obaidi, Ibrahim K Al Abid, Abdullah Almazouni, Mohammad Saif, Habibulah Abdullah, Mahmoud Alothman Agha, Ashraf Mahmoud

**Affiliations:** 1 Medicine, Tawam Hospital, Al Ain, ARE; 2 Medicine, Ras Al Khaimah Medical and Health Sciences University, Ras Al Khaimah, ARE; 3 Cardiology, Mediclinic Al Ain Hospital, Al Ain, ARE; 4 Medicine, Al Qassimi Hospital, Sharjah, ARE; 5 Internal Medicine, Mediclinic Al Ain Hospital, Al Ain, ARE; 6 Medicine, Burjeel Hospital, Abu Dhabi, ARE

**Keywords:** artificial intelligence, deep learning, developmental dysplasia of the hip, radiographic diagnosis, ultrasound imaging

## Abstract

The field of pediatric musculoskeletal imaging is evolving at the moment because of artificial intelligence (AI) and is starting to play an important role in diagnosing developmental dysplasia of the hip (DDH), a common pediatric orthopedic condition that can lead to gait problems, functional issues, pain, and early-onset osteoarthritis if not treated early. Standard diagnostic methods depend on clinical examination and imaging systems, including ultrasound and radiography, that are highly operator-dependent and susceptible to measurement error. Recent developments in AI, including deep learning and convolutional neural networks, have enabled automated image analysis, detecting anatomical landmarks automatically, and accurate measurement of the alpha angle, beta angle, and acetabular index as important diagnostic parameters.

This article presents a literature review that summarizes the current literature in the field of AI-assisted DDH diagnosis using ultrasound and radiographic imaging. In the literature, AI-based models exhibit great diagnostic accuracy, better consistency, and lower interobserver variability than traditional evaluation. Advanced architectures, such as segmentation networks and 3D convolutional models, further improve image quality assessment and classification. Portable ultrasound systems and cloud-based diagnostic platforms powered by AI provide hope for expanding access to DDH screening in low-resource settings.

These advances are, however, difficult because of dataset heterogeneity, the generalizability of deep learning models across different people and imaging devices, and the interpretability and integration of deep learning models into clinical workflows. Long-term multicenter validation, standardization in reporting, and regulatory control are necessary to guarantee safe clinical translation. The literature generally endorses AI as a useful supplement to the clinician's clinical toolkit; its utilization could potentially offer better early, accurate, and scalable diagnosis of DDH.

## Introduction and background

Artificial intelligence (AI) revolutionizes medicine today, affecting its diagnosis, treatment, and prediction in several medical fields. The development of fast computation, access to information, and algorithmic complexity has allowed AI systems to treat sophisticated medical information more and more accurately than humans are capable of. Among these advances, methods that use deep learning have so far delivered the best potential to improve medical imaging accuracy, where it is essential to have the best pattern search, quantitative assessment, and feature extraction.

Developmental dysplasia of the hip (DDH) is a common orthopedic condition that is characterized by an atypical growth of the femoral head and the acetabulum. If diagnosis is delayed, patients can suffer from ongoing joint instability, mechanical changes, pain that could affect the patient’s quality of life, and also early-onset osteoarthritis. Consequently, early diagnosis is important because DDH requires early treatment to prevent undesirable outcomes as well as decrease the need for surgical intervention. The diagnostic strategies for DDH are based on clinical examination, along with imaging (ultrasound and radiography). These approaches are highly operator-dependent and technically advanced. However, inaccuracies from image acquisition, interpretation, and measurement of anatomical landmarks may result in differential diagnosis, especially in marginal or subtle cases. In settings where the opportunity or access to professional sonographers or pediatric radiologists is limited, these challenges are also exacerbated.

There have been recent advances in AI and deep learning, which have opened new avenues to compensate for these limitations. AI-guided technologies assist in automating image processing, standardizing measurements, and assisting clinicians with decision-making. It can also help to minimize operator involvement and increase reliability, as well as boost early diagnosis, and in turn assist large-scale screening programs and increase the quantity of reliable DDH analysis.

## Review

Methodology 

This manuscript is a narrative literature review synthesizing peer-reviewed evidence on AI applications in pediatric hip imaging for DDH. Literature was identified through targeted searches and citation-chaining of key papers to capture influential and recent work relevant to DDH imaging tasks (e.g., detection/classification and measurement). The review emphasizes studies with sufficient methodological detail and clinically interpretable outcomes; non-peer-reviewed items, opinion pieces, and reports lacking extractable or DDH-specific imaging information were not emphasized. Study characteristics were summarized using a structured internal checklist (imaging modality, dataset/population, AI approach, reference standard, reported performance metrics, and limitations). Because of heterogeneity in datasets, model types, and outcome reporting, findings were synthesized qualitatively by organizing studies by imaging modality and clinical task and comparing patterns across themes (performance, validation/generalizability, feasibility, and limitations) rather than pooling results quantitatively.

Results 

AI Techniques and Algorithms in Diagnosing DDH

Recent advances in convolutional neural networks (CNNs) and other AI systems have had a big impact on the analysis of medical images to help in diagnosing DDH. It has been well recognized that using CNN-based methods is a popular method for automatic segmentation and categorization of hip ultrasound images. A salient example is Hareendranathan et al., who proposed a CNN-based framework for the accurate segmentation of the acetabulum using multi-scale superpixels as input to an AlexNet architecture, which enabled accurate segmentation for the acetabulum [1].

In addition to two-dimensional CNNs, three-dimensional convolutional neural networks (3DConvNets) have been explored for video-based image analysis. Architectures such as C3D and I3D have demonstrated strong performance in classification tasks. The C3D network, characterized by a uniform architecture of 3 × 3 × 3 convolutional kernels, achieved an accuracy of 52.8% on the UCF101 dataset. The I3D model, which integrates both 2D and 3D convolutions within a two-stream framework and is pretrained on the Kinetics dataset, demonstrated substantially higher accuracies, achieving 80.9% on the human motion database (HMDB)-51 dataset and 98.0% on the UCF-101 dataset [1].

Various more sophisticated segmentation architectures, such as 3D U-Net, 3D V-Net, and 3D deeply supervised networks, have also been implemented for video segmentation of images. However, it is difficult to apply these models to 3D ultrasound volumes of the hip, primarily due to the scarcity of large, well-annotated datasets needed for proper training and validation [1]. The range of imaging modalities, AI techniques, and diagnostic tasks identified across the reviewed studies is summarized in Table 1.

**Table 1:** Summary of AI applications in DDH diagnosis by imaging modality CNNs: Convolutional neural networks, DDH: Developmental dysplasia of the hip, AP: Anteroposterior

Imaging modality	AI techniques applied	Primary diagnostic tasks	Key outcomes reported in reviewed studies
Ultrasound	CNNs, 3D CNNs, segmentation networks (e.g., U-Net variants)	Image quality assessment, acetabular and femoral head segmentation, alpha and beta angle measurement	Improved diagnostic consistency, reduced operator dependence, accurate landmark detection, and automated quality assessment
Ultrasound (video/3D)	3D convolutional networks, spatiotemporal models	Frame selection, scan adequacy assessment, and dynamic landmark recognition	Enhanced classification accuracy, improved reliability in variable image quality
Radiography (AP pelvis)	CNNs, multi-task learning networks	DDH classification, severity assessment, acetabular index, and angle measurements	Diagnostic accuracy comparable to expert clinicians, reduced interobserver variability
Multimodal Imaging	Integrated deep learning frameworks	Combined imaging analysis and clinical decision support	Increased sensitivity for borderline and mild DDH cases, improved diagnostic confidence
Portable/point-of-care ultrasound	AI-assisted automated analysis	Screening and preliminary diagnosis	Expanded access to DDH screening, particularly in low-resource settings

Practical Deployment and Dataset Training

Many deep learning models used towards the diagnosis of DDH are typically trained on hip ultrasound images taken by competent sonographers of good quality. These images were taken to obtain such vital anatomical features as a straight and horizontal acetabulum and a round, formed, and defined femoral head. The quality of training data is usually assessed by expert clinicians so as to ensure diagnostic reliability and clinical relevance [1].

To meet the requirement to assess objectively the efficacy of the quality of the information provided, a new three-dimensional CNN model was developed, which evaluates independently the suitability of hip ultrasound images for the diagnosis of DDH. The proposed model has been tested with two large clinical data sets. The primary dataset consisted of 2,187 hip ultrasound scans, the first large-scale validation study investigating automated assessment of hip scan quality [1]. Systematic model predictions were also analyzed using the performance across patient subgroups according to age (zero to three months and >3 months) and sex (male and female) [1].

In another dataset including 107 hip ultrasound images, a multi-reader study was carried out where four evaluators assessed scan quality based on a 10-point semi-quantitative scoring system. The AI-based algorithm's predictions were compared against the scores of the evaluators. The AI system was able to distinguish diagnostically adequate or less than adequate scans consistently and improved accurate and consistent diagnoses, decreasing dependency on subjective human judgment [1].

AI-assisted Radiographic Analysis

Convolutional neural networks are also used for the automatic calculation of radiographic measurements for DDH diagnostic purposes. In one dissertation-derived study, a CNN model was trained over an average of 1,500 radiographic images. Although the model performed well on high-contrast images, it showed lower accuracy on low-contrast images. Our findings suggest that although CNNs have considerable promise for enhancing AI-based DDH diagnosis and improving the diagnostic process, image quality was a key driving factor [2].

Artificial intelligence-assisted imaging technologies have shown very satisfactory diagnostic performance for DDH and have the ability to improve diagnostic accuracy provided by established imaging standards such as X-ray and ultrasound. Some of the CNN-based models have shown the most significant advantages in DDH-assisted diagnosis among the different AI methods [3]. Radiography remains the primary imaging modality for DDH assessment concerning the anteroposterior pelvis [4].

Zhang et al. analyzed 10,219 anteroposterior pelvic radiographs between 2014 and 2018 in a wide retrospective study. In addition, each radiograph was assigned a label according to diagnostic criteria by physicians. Dislocation and subluxation (dislocation) were classified into the first group of images, while normal hips and acetabular dysplasia were the second (non-dislocation) group [5].

A deep learning system was trained using 9,081 radiographs and subsequently tested on 1,138 radiographs to compare its diagnostic performance with that of human clinicians [5]. Detection of DDH was evaluated using receiver operating characteristic (ROC) curve analysis. Bland-Altman plots were also used to evaluate the agreement between AI-synthesized acetabular index measurements and assessments obtained from radiologists [5].

Diagnostic Accuracy

With the use of AI, mainly deep learning models, computerized analysis of medical imaging has enhanced diagnostic accuracy for patients with suspected DDH. A study that used a multi-task hourglass network to evaluate DDH and read radiographs of the same cohort, not only diagnosing DDH but also assessing the severity of the condition. The AI model was more sensitive and specific than manual assessment of radiographs, especially when fast recognition was essential in clinical practice. These findings prove that AI could help in avoiding human errors and improve the detection process [6]. Another study, screening using AI systems based on innovative algorithms, assessed hip joint alignment with the help of pelvic radiographs. The ability of the system not only expedited interpretation of images but also provided similar degrees of diagnostic specificity as could not be achieved easily with human intervention, especially in complicated or borderline cases [4].

In Sha et al.'s study, a machine learning or AI‐guided diagnostic technique was designed to bridge imaging with clinical information to enable detailed diagnosis of DDH. This system used CNNs for the analysis of X-ray and ultrasound images to achieve extremely accurate diagnostic resolution. Notably, the model was shown to detect subtle DDH traits not commonly diagnosed by healthcare professionals, improving accuracy and sensitivity in the early and mild phases of dysplasia [7]. The study investigated important clinical thought-based diagnostic software for DDH. It combines various pieces of data such as imaging, history of the patient, and physical examination results. The AI model increased the confidence for diagnosis, minimized misdiagnosis, and provided decision support in real time, as it could support better diagnosis with clinical judgment [7]; therefore, the use of AI alongside clinical judgment is a necessity to provide an accurate and timely diagnosis.

Finally, the significance of AI in an autonomous diagnostic system was highlighted in a study that focused on a novel DDH screening approach. Machine learning algorithms were able to identify DDH accurately since they compared hip joint structures on typical anteroposterior radiographs with a large database of known cases. In particular, the findings imply that such AI-based screening techniques can reach higher diagnostic accuracy, especially in areas with limited access to experienced radiologists [8].

Data Sources and Training Datasets

As pointed out in several studies, high-quality, well-annotated datasets are vital in the training of AI models for DDH diagnosis. One dataset comprising 1,723 pelvic X-ray images was taken at Peking University Third Hospital between 2017 and 2021. The images were annotated by three orthopedic specialists, who annotated key anatomical landmarks, including the center of the femoral head and the margins of the acetabulum. Measurements also included the center-edge (CE) angle and Tönnis angle. The dataset was divided into training (1,533 images), testing (150 images), and evaluation datasets, and diagnostic accuracy was evaluated using the Crowe classification system [8,9].

Another study had 921 high-quality ultrasound images for screening for DDH using AI to extract information and to identify anatomical structures such as the femoral head and acetabular roof. Images with sufficient visualization and a consistent scanning angle within ± 5° were included to ensure data integrity and to measure alpha and beta angles to the accuracy of experienced clinicians [10].

Open-access imaging repositories have also been shown to be useful sources and facilitators for the development of AI-based models in healthcare. Datasets such as The Cancer Imaging Archive (TCIA) are also available to assist AI training and enable meta-information to be applied to several imaging technologies. These repositories are potentially adapted toward hip pathology, integrating DDH imaging data in order to build AI models that reflect the patient range [10].

Artificial intelligence has additionally increased the efficiency of ultrasound technology by assisting in reducing operator dependence and increasing diagnostic accuracy. Some recent publications have suggested that AI-based analysis of high-quality ultrasound images will provide a more accurate diagnosis. While some studies have concentrated on respiratory disease diagnosis with lung ultrasound, similar AI methods have been successfully applied to musculoskeletal imaging, such as DDH diagnosis. This highlights the opportunity that AI has in translating ultrasound imaging into a more objective and consistent diagnosis [11].

Discussion

The results of the present review suggest a considerable application of artificial intelligence for the diagnosis of developmental dysplasia of the hip by improving diagnostic accuracy, efficiency, and consistency between imaging modalities. Deep learning-driven systems, especially convolutional neural network systems, have been demonstrated to analyze both ultrasound and radiographic images and achieve a level of accuracy comparable to expert clinician interpretation. The principal advantages and limitations of AI-assisted DDH diagnosis identified in the reviewed literature are outlined in Table 2.

**Table 2:** Reported advantages and limitations of AI-assisted DDH diagnosis DDH: Developmental dysplasia of the hip

Domain	Reported advantages	Reported limitations
Diagnostic accuracy	High sensitivity and specificity; reliable detection of subtle DDH features	Reduced accuracy with low-quality or low-contrast images
Measurement consistency	Automated, standardized measurement of acetabular index, alpha, and beta angles	Dependence on precise landmark visibility
Operator dependence	Reduced reliance on expert sonographers or radiologists	Continued need for expert oversight in atypical cases
Generalizability	Effective performance in controlled datasets	Limited performance across different populations, age groups, and imaging systems
Workflow Integration	Faster analysis, decision support, scalable screening	Barriers to implementation, training requirements, and interoperability issues
Interpretability	Objective image-based outputs	Deep learning 'black-box' behavior affecting clinician trust
Access to care	Improved screening availability in underserved settings	Infrastructure and regulatory challenges in implementation

The enhanced sensitivity is critical in early or mild forms of DDH, with clinical manifestations that might be imperceptible with subtle imaging abnormalities [7]. Clinical thought-based AI software also showed that the integration of algorithmic analysis along with clinical reasoning can increase diagnostic confidence, minimize misdiagnosis, and support real-time decision-making, consistent with the theory that AI needs to serve as an adjunct rather than a substitute approach to clinician expertise [7].

Training alone on large datasets of radiographic images has been effective in better identifying DDH than traditional diagnostic methods using AI-based screening systems that use only the available data. These systems are likely to have the greatest impact in resource-limited settings in which access to experienced radiologists is limited, as the problem of late and missed diagnoses is a major issue [8]. The AI-augmented screening may help to reduce long-term morbidity from surgery, based on improved early diagnosis.

However, there are some challenges and limitations that need to be considered before achieving broad clinical application. The dependence of AI models on good-quality, meticulously annotated data is one of the biggest drawbacks cited in studies. Poor training data performance can be harmful to the implementation of algorithms in populations/imaging environments where those represented in the training data are not the same. Differences in ultrasound image quality, for instance, are documented to directly influence diagnostic accuracy, lower-quality images resulting in decreased sensitivity and increased rates of misclassification [11].

Generalizability continues to be a challenge for AI systems in DDH diagnosis. Although many models work well in their training populations, they tend to struggle to produce accurate results when extended to other populations, age groups, or new imaging techniques. Variability in patient anatomy, imaging protocols, or radiographic systems could be important factors that are difficult for AI models to consider. A performance can also be affected by the type of radiographic equipment utilized, which is further reinforced by studies for diverse and representative training data sets and training of trained AI models [12].

A further concern is the interpretability and transparency of deep learning models. Many describe convolutional neural networks as 'black box' systems due to their internally very complex decision-making processes. Such a lack of transparency would erode clinician trust, especially if diagnostic decisions are not evident or in conflict with clinical judgment. The lack of clarity in interpreting how AI systems reach their conclusions represents a barrier to adoption, particularly in sophisticated or atypical DDH cases [6].

The AI systems have also shown limitations in addressing challenging or atypical presentations of DDH. While these models are successful in capturing general patterns, they tend to have some difficulty regarding exceptional alterations in anatomy or borderline abnormalities that call for sophisticated clinical recognition. For instance, deep learning architectures that use YOLOv5 (Ultralytics, Frederick, MA, USA) modelologies have less sensitive performance on cases due to mild deformities of the joint, indicating the continual need for expert clinical oversight in difficult cases of diagnosis [13]. Upcoming research into an AI-assisted DDH diagnosis needs to deal with these shortcomings.

It is already well established that the accuracy of DDH diagnosis with AI-assisted systems is very high, and it is expected that further improvements will be made as algorithms develop. The AI-based architectures are found to reach the level of human-like accuracy in recent studies [14-16]. Thus, AI imaging could assist in earlier diagnosis, more immediate intervention, and lower diagnostic variability due to the human operator.

Multimodal integration is promising for further advancement in the future. Such a combination of ultrasound, radiography, and magnetic resonance analysis data would enable AI systems to obtain more holistic diagnostic summative assessments and to offer assistance to patients during the actual image acquisition and interpretation. With such integration, diagnostic accuracy and workflow effectiveness may be enhanced [14]. Also, AI-based personalized risk assessment can lead to personalized diagnostic and treatment approaches, which may further improve patient outcomes as the diversity of cases is extended.

Explainable artificial intelligence (XAI) is also an important aspect of future consideration. The goal of XAI is to improve the interpretable nature and clarity of the AI decision-making processes for clinicians, increasing trust and facilitating both their clinical translation and adoption. It could be used to play a key role in the integrated development of DDH diagnostic systems with a significant impact on the future application of AI-assisted imaging as part of routine pediatric practice [17]. The emerging technology of AI-supported wearables could also have a big impact on DDH care in the future. Wearable devices may make for passive monitoring or passive observation of high-risk infants for DDH and enable clinicians to make actionable observations about these infants. Advancements like that would lead to a broader, more extensive spectrum of early diagnosis and monitoring [18]. Also, due to the continuous evolution of new data, improvements over algorithms, multicenter validation algorithms, etc., these innovations can reform DDH diagnosis in terms of improved accuracy, reliability, patient focus, and user sensitivity.

Regulations and Guidelines

The literature reviewed shows the importance of regulation and a unified set of standards in the safe and effective use of AI for hip developmental dysplasia detection and diagnosis. A study demonstrated evidence where FDA-supported software (MEDO-Hip; Medo.AI, Edmonton, CAN) was applied to a systematic clinical workflow for DDH screening, highlighting the necessity of compliance with regulatory standards in medical AI applications [19]. This reflects the approach prescribed by professional associations such as the American College of Radiology, which highlights using AI tools verified by researchers and controlled to conform to the norms of practice as well [19].

Ethical issues were dealt with in detail with strict adherence to informed consent; parental consent was obtained prior to infant scanning, and the study was approved by a Health Research Ethics Board. Imaging protocols were based on existing clinical guidelines, with ultrasound being done between four and sixteen weeks of age [19]. Quality assurance was implemented through internal follow-up processes to improve referral accuracy as well as through the programming of AI tools to only accept scans with essential anatomic landmarks [19].

The AI-assisted screening training instructions were explicit and included video tutorials, PowerPoint-generated tutorials, and supervised scanning over two to four days. As the user’s competence improved, unsupervised scanning was implemented. Data confidentiality was ensured by limiting access to the data as per the institutional protocol [19]. While the above-mentioned protective factors were designed, additional prospective studies will be needed to assess the long-term safety and clinical utility of AI-assisted imaging technologies on pediatric patients and to verify their use in early DDH diagnoses [3].

Regulations for AI healthcare frameworks are ongoing. Meanwhile, the FDA has a pre-market approval mechanism for AI medical devices, focusing on clinical validation, algorithmic transparency, and post-market monitoring [20]. And the European Union's Medical Device Regulation requires strict safety and performance expectations for AI tools, such as continuous post-market surveillance [21]. The ethical deployment of AI in healthcare has also been backed up by international organizations, such as the WHO, which argues for fairness, accountability, transparency, and patient safety in all AI healthcare applications [22]. Overall, these regulatory actions seek to align the technological and patient protection balance to guarantee that the AI tools used for DDH detection will be effective and trustworthy.

User Acceptance and Training in AI Detection of DDH

The accurate diagnosis of DDH is dependent on acquiring high-definition ultrasound images, for which expertise in technology is required [23]. Automated AI systems for DDH detection are highly sensitive to image quality, sometimes even more than human experts. Poor-quality images, typically scoring ≤7 out of 10, are more likely to be misinterpreted or require clarification from a human [23].

Therefore, Hareendranathan et al. designed a study to provide a structured 10-point scoring system for scan quality, along with the scan quality associated with six imaging variables that characterize Graf analysis. This system has a score for poor (2/10), moderate (6/10), or excellent (10/10) scans, showing a greater reliability compared with holistic scoring (inter-rater correlation coefficient of 0.93 vs 0.68) [23]. Hence, a scoring system can be used as a step of preprocessing before AI-based diagnosis, allowing low-quality scans to be flagged for repeat acquisition and only diagnostically adequate images to be analyzed [23]. The AI systems may further help users enhance image accuracy in response to real-time feedback. Raising awareness in radiologists and sonographers about how to work with scan quality scoring systems and feedback from AI might help improve diagnosis [23].

Future directions are in the reduction of observer variability by computerized measurement of image properties and validation of such systems with larger and more heterogeneous clinical populations. User acceptance is still the most important determinant of effective AI adoption. That is, evidence indicates that clinicians will be more willing to use AI technologies if they are engaged in the development process and if AI technologies are designed to assist, rather than replace, their clinical skills [24]. It is suggested that training programs providing explicit information are another way to build trust and confidence among users, as they are aware of the pros and cons of AI systems (especially the problem of image quality dependence) [25]. Interdisciplinary collaboration between AI developers and health care professionals also ensures that AI tools are intuitive, relevant in clinical practice, and in line with diagnosis needs in the real world [26]. Continuous learning, performance feedback, and real-time analytics techniques can benefit clinicians in improving imaging capabilities and further utilizing AI with enhanced clinical practice.

Cost Effectiveness

In spite of the advantages, early implementation challenges were noted, such as technical problems, the need for training, and infrastructure [27]. Service providers expressed a positive view on the quality of the AI-assisted instruments, with high satisfaction due to the simplicity of use and the fact that direct information and test results were accessible rapidly, which helped to enhance clinical confidence and to decrease patient travelling for follow-up visits. These findings indicate that AI-based DDH diagnostic systems might be an economical option for early DDH screening if implemented with proper support and approaches, especially in rural or deprived areas [27]. Also, AI applications, including automated diagnostics, virtual assistants, and medical robotics, have also been found to be less labor-intensive and efficient in workflow by reducing human involvement [28,29]. Early detection of the disease via AI use may be useful in lowering unnecessary procedures and costs by increasing the diagnostic accuracy [30]. Moreover, communication, documentation, and healthcare errors are optimized to deliver the best healthcare process [31]. Collectively, these variables underpin the economic potential and pragmatic issues to navigate if AI is to enter widespread clinical practice.

Interdisciplinary Collaboration

The effective application of AI in DDH diagnosis requires an interdisciplinary team of colleagues across healthcare professionals (clinicians, engineers, informaticians, and regulatory specialists). Orthopedic surgeons and radiologists are pivotal for the identification of important anatomical markers, for example, acetabular angles and femoral alignment, and in ensuring the correct clinical significance of their imaging datasets. Specifically, radiologists provide contributions to the quality assurance of images and the pre-labeling process for datasets employed in AI training, ensuring that algorithmic output approaches clinical expectations.

Deep learning AI engineers build algorithms for DDH diagnosis, including convolutional neural networks, as well as models, such as Mask region-based (R)-CNN, for segmentation accuracy and landmark detection. Understanding differences in visual variability and population diversity, these engineers improve the reliability and the generalizability of AI systems on heterogeneous datasets [32,33].

Integrating AI into clinical practice, health informatics specialists ensure interoperability with electronic health records and imaging databases with compliance to Digital Imaging and Communications in Medicine (DICOM) standards. They help streamline clinical workflows, make AI technologies easier to use, and support the successful implementation of such technologies in practice [34]. The AI systems comply with healthcare regulations and data protection requirements under regulations like the General Data Protection Regulation (GDPR) and the Health Insurance Portability and Accountability Act (HIPAA); thus, regulatory and ethical professionals ensure compliance. They also discuss the ethics of dataset bias, fair access to AI technologies, and obtaining patients’ informed consent for research and clinical uses of patient data.

Pediatricians and public health experts are involved in the initial stages of AI-based screening programs that are focused on early assessment and prevention, especially in high-risk populations. Where pediatricians consider the needs of individual patients, public health scientists are proposing scalable and inclusive screening models to make AI solutions applicable and accessible more broadly within various treatment systems [32,33].

## Conclusions

Artificial intelligence has demonstrated remarkable potential to diagnose DDH. Deep learning with CNNs and 3D imaging techniques has recently been developed for automatic ultrasound and radiographic image examination with diagnostic accuracy up to the level of experienced clinicians. The AI systems have already proven that they can accurately measure important diagnostic variables while at the same time minimizing variability among observers and providing uniformity in the distribution of heterogeneous imaging quality. The implementation of AI for DDH screening and diagnostic workflows brings significant clinical advantages in terms of early detection, enhanced efficacy, and better accessibility of care in specific resource-limited regions. Evolving systems, such as handheld ultrasound devices, cloud-based diagnostic systems, explainable AI frameworks, and wearable technologies, also support the evolving function and are an integral part of using AI in pediatric orthopedic practice. However, limitations such as data quality, generalizability, interpretability, workflow integration, and regulations remain an unsolved issue. To overcome these limitations, continuous multicenter research is needed. Based on the literature, we can safely say that AI-assisted diagnostic tools are a promising field for standardizing and cost-effectively diagnosing DDH, improving patient outcomes, and limiting the long-term burden of the condition.
